# Screening for Novel *LOX* and *SOD1* Variants in Keratoconus Patients from Brazil

**DOI:** 10.18502/jovr.v15i2.6730

**Published:** 2020-04-06

**Authors:** Diego Nery Benevides Gadelha, Alex Felipe Barbosa Feitosa, Rafaela Gomes da Silva, Luana Talita Antunes, Matheus Cavalcanti Muniz, Matheus Alencar de Oliveira, Dáfine de Oliveira Andrade, Nathalia Mayanna da Paz Silva, Sebastião Cronemberger, Bruno Luiz Fonseca Schamber-Reis

**Affiliations:** ^1^ Department of Medical Genetics, School of Medical Sciences, UNIFACISA, Campina Grande, Paraíba, Brazil; ^2^ Medical School, University of Minas Gerais, Belo Horizonte, Brazil

**Keywords:** Keratoconus, Mutation, LOX, SOD1

## Abstract

**Purpose:**

To investigate the presence of the variants of lysyl oxygenase (*LOX*) and superoxide dismutase 1 (*SOD1*) genes in Brazilian patients with advanced keratoconus.

**Methods:**

Donor genomic DNA extracted from blood samples was screened for 5'UTR, exonic *LOX, *and *SOD1* variants in a subset of 26 patients presenting with advanced keratoconus (KISA > 1000% and I–S > 2.0) by Sanger sequencing. The impact of non-synonymous amino acid changes was evaluated by SIFT, PMUT, and PolyPhen algorithms. The Mutation Taster tool was used to evaluate the potential impact of formation of new donor and acceptor splice sites in the promoter region of affected volunteers carrying sequence variants. A 7-base *SOD1* deletion (IVS2 + 50del7bp) previously associated with keratoconus was screened in 140 patients presenting classical keratoconus by gel fragment analysis, and positive samples were sequenced for confirmation.

**Results:**

We found an unreported missense variant in *LOX* exon 6 in one heterozygous patient, leading to substitution of proline with threonine at residue 392 (p. Thr392Pro) of *LOX* protein sequence. This mutation was predicted to be potentially damaging to *LOX* protein. Another *LOX* variant, Arg158Gln, was also detected in another patient but predicted to be non-pathogenic. Two additional new polymorphisms in *LOX* 5'UTR region (–116C > T and –58C > T) were found in two patients presenting with advanced keratoconus and were predicted to modulate or create donor/acceptor splice sites in *LOX* transcripts. Additionally, *SOD1* deletion was detected in one patient presenting with severe keratoconus, not in control samples.

**Conclusion:**

We described three novel LOX polymorphisms identified for the first time in Brazilian patients with advanced keratoconus, as well as a previously described *SOD1* deletion strongly associated with keratoconus. A possible role of these variants in modulating transcript levels in the cornea of affected individual requires further investigation.

##  INTRODUCTION

Keratoplasty is the most frequent tissue transplantation in Brazil, comprising 60% of all organ and tissue transplantations in the country.^[1]^ Among these, around 50% of all performed keratoplasties are due to keratoconus (KC), which is characterized by corneal ectasia and stromal protrusion in a conical shape. Clinical signs of KC usually manifest first at puberty and in young adults leading to visual deterioration due to irregular astigmatism, myopia, and corneal scarring in about 20% of patients.^[2,3]^ Although KC manifests in only one eye in most patients, the disease can progress to bilaterality in severely affected individuals.^[4]^ Histologically, KC is characterized by impaired collagen secretion resulting from degeneration of keratocytes as well as by epithelial and stromal changes leading to disorganization of fibers involved in tissue resistance and stability.^[5]^ KC incidence is estimated at 1/2,000 individuals in the general population, with a prevalence of 50 to 230 cases/100,000 in Western countries.^[6]^ Diagnosis is usually based on qualitative corneal topography and quantitative videokeratography of posterior corneal surface confirmed by a KISA index > 100%.^[7]^ Refractive error in KC can be corrected with glasses or contact lenses. About 20% of patients have more advanced disease, for which Ferrara intrastromal corneal ring segments (Ferrara rings) are recommended, along with penetrating keratoplasty in most cases.^[6,8]^

The etiology of KC is not completely understood. The disorder has been described as multifactorial and polygenic.^[9,10,11]^ Most cases are sporadic suggesting the role of environmental factors in susceptible individuals, including atopic factors, mechanical trauma, and ultraviolet (UV) light exposure, which may promote corneal thinning and tissue degradation. However, a strong association of isolated environmental factors with KC has not been effectively demonstrated.^[4,10,12]^ Twins^[13]^ and family-based studies,^[14]^ candidate gene strategies, and genomic association studies have shown the influence of DNA variants on KC development. Many candidate genes regulating production or arrangement of collagen fibers,^[15,16,17]^ keratocyte proliferation/apoptosis,^[12]^ morphogenesis, and regulation of oxidative stress,^[18,19]^ as well as chromosomal *loci* bearing specific single nucleotide polymorphisms (SNPs) have been described in KC patients. The *LOX *gene, located at 5q23.1, is among the most investigated genes in KC.^[15,16,20]^ *LOX* encodes a copper-dependent, oxido-deaminase lysyl oxidase enzyme, which plays a role in extracellular matrix maintenance by oxidizing lysine residues and promoting the formation of covalent cross-links of collagens and elastin,^[16]^ making them insoluble in the extracellular environment.^[21]^ Reduced *LOX* activity has been detected in the cornea of patients with KC, suggesting a significant role of this gene in the pathogenesis of disease.^[15,22,23]^ Two of the main missense mutations, Arg158Gln (rs1800449) and Pro159Gln (rs41407546), have been described in both Italian and Iranian populations^[16,20]^. A role of the Arg158Gln mutation in KC has been demonstrated in a genome-wide association study that also identified an SNP associated with the 5'UTR region.^[20]^ These data suggest a likely role of the *LOX *gene in the development of KC. Another candidate gene is *SOD1* (superoxide dismutase 1), which has a direct involvement in antioxidative processes associated with reactive oxygen species (ROS) elimination and reduction of oxidative stress that is increased in the corneal cells of patients with KC.^[24]^ Located at 21q22.11, *SOD1 *encodes a copper and zinc-dependent cytoplasmic enzyme associated with dismutation of ROS such as superoxide radicals.^[25]^ There is evidence of a correlation between *SOD1* variations and the development of KC, given the presence of high levels of oxidative stress markers and/or accumulation of these markers in the cornea of affected individuals.^[26]^ Several studies have described a heterozygous genomic 7-base deletion in the intron 2 of the *SOD1* gene (IVS2 + 50del7bp) in the US population which significantly affected the conformation and function leading to an increase in oxidative stress in the cornea.^[27,28,29]^ Two sporadic carriers of this deletion were also detected in the Italian population,^[30]^ and nine in the Greek population.^[29]^ The involvement of *SOD1* in other ocular diseases such as primary open-angle glaucoma has also been described.^[31]^

Although already well studied,^[32,33]^ the genetic basis of KC is still poorly understood.^[34]^ Despite the availability of data on the involvement of *SOD1* and *LOX* mutations in the development of KC in specific cohorts, little information is available regarding Brazilian patients. A single study, using a gene candidate approach, showed three non-synonymous substitutions (Leu68His, Arg131Ser, and Asp105Glu) in *VSX1* gene segregating with the phenotype in patients from the Northeast of Brazil who presented with sporadic KC, suggesting a role for this gene in KC development.^[35]^ However, no additional studies are available in Brazil regarding polymorphisms in other genes whose involvement in KC has already been described. The implication of *SOD1* and *LOX* mutations on KC development may be important risk factors for the disease and their involvement in disease pathogenesis depends on additional studies on different ethnicities.^[25,33]^ Considering that this pathology is considered a major public health issue in Brazil, the aim of this study was to investigate the presence of genetic variants in *SOD1* and *LOX *genes in individuals affected by advanced KC.

##  METHODS

### Study Protocol and Participants

The research protocol was approved by the Human Research Ethics Committee at Unifacisa (CAAE 98902918.6.0000.5175) as determined by Resolution 466/12 of the Brazilian National Health Council and the Declaration of Helsinki. All participants signed an informed consent form prior to their enrolment.

Volunteer participants were recruited at an ophthalmology outpatient clinic (UNIFACISA/Campina Grande) in the state of Paraiba, Brazil. The sample included residents of Campina Grande and neighboring cities (Lagoa Seca, Queimadas, Esperança and Massaranduba). At the time of enrolment, 10 mL of whole blood were collected for later DNA extraction and stored in a –80°C freezer at the Unifacisa Medical Genetics Core Laboratory (NUGEM). Participants were included if they met minimal topographic criteria for the diagnosis of KC: KISA > 100% and inferior–superior asymmetry (I–S) ratio > 1.4 diopters (D) in at least one eye.^[4]^ Diagnostic confirmation was based on keratometry, performed in an Eyetech CT2000 SLE topographer (Eyetech Equipment, São Paulo, Brazil) and Schwind Sirius Scheimpflug (Eye-tech-Solutions, GmbH&Co. Kleinostheim, Germany) tomograph. Patients with other eye disorders (glaucoma or cataract) or those undergoing corneal transplantation were excluded.

Of the overall group consisting of 140 patients, 26 patients had KISA > 1000% and I–S > 2.0; these were included in an advanced KC subgroup. To maximize the chance of detecting the variables with the highest pathogenic potential, DNA sequencing for *LOX* gene was performed specifically for this subgroup. A control group of 50 healthy subjects was also screened.

### DNA Sequencing

Genomic DNA was extracted from blood samples. All *SOD1* and *LOX* gene exons were sequenced after amplification by polymerase chain reaction (PCR). For that, appropriate forward and reverse primers were used as previously described [29,36, Supplementary  Table 1] . The primers used for amplification of *SOD1 *exon 1 also spanned the proximal *5' *untranslated region (UTR), corresponding to approximately 150 base pairs (bp) upstream from *SOD1* start codon.

PCR was run in 25 µL volumes containing 12.5 μL GoTaq Green Master Mix (Promega, USA), 5 pmol of each oligonucleotide primer, and 1 μL resuspended DNA solution at an approximate concentration of 200 ng/μL. Reactions were performed in a thermocycler as follows: 5 min at 95°C, followed by 35 cycles (1 min at 95°C for DNA denaturation, 1 min for primer annealing, and 1 min at 72°C for extension), and a final 5 min extension step at 72°C. Specificity and quality of the amplicons were assessed in agarose gel at 0.8% containing ethidium bromide (0.5µg/mL). Fragments were visualized by UV light using a LPIX transilluminator (Loccus Biotecnologia, Brazil).

To sequence PCR products, amplified exon segments were purified using the Wizard SV Gel and PCR Clean-up System (Promega, USA). Purity was assessed using a Nano-volume spectrophotometer (NanoVue Plus, GE), considering a 260 nm/280 nm absorbance ratio of 1.7 to 2.0 as the purity standard. Each sequencing reaction (7.5 μL final volume) contained 10 ng per 100 bp of specific exons and 10 pmoles of each individual primer. Reactions were submitted to Myleus Biotechnology (Belo Horizonte/MG, Brazil) for Sanger sequencing on an ABI3130 platform. Analysis of the electropherograms was performed using Sequence Scanner 2 v.2.0 (Applied Biosystems, EUA) and Sequence Viewer v.7.0.2 (CLCBio, Denmark) software. Gene sequence readings were compared to ancestral *SOD1* (ENSG00000142168) and *LOX* (ENSG00000113083) sequences downloaded from the Ensemble genome browser (www.ensembl.org). All variations detected in samples were reconfirmed by a new DNA extraction and sequencing.

To test for the presence of the intronic 7-base deletion IVS2 + 50del7bp (c.169+50delTAAACAG) in *SOD1*, samples were tested by PCR in 140 KC positive patients and 120 controls, using previously described amplification conditions.^[29]^ Detection of this deletion in patients involved direct visualization of a 211 bp amplicon in a 20 x 20 cm PAGE (5%) electrophoresis, followed by confirmatory DNA sequencing of positive samples.

### Prediction of Pathogenic Potential of Gene Variants

To predict the impact of nonsynonymous substitutions in *LOX* protein variants, we used SIFT (http://sift.jcvi.org/),^[37]^ PolyPhen2 (http://genetics.bwh.harvard.edu/pph2/), and PMUT (http://mmb.pcb.ub.es/PMut/) tools.^[38]^ These algorithms assigned scores (i.e., probability of the substitution being damaging) ranging from 0 to 1. Scores are generated through comparison between the ancestral amino acid sequence and the variant sequence (missense mutation). In the SIFT platform, a score up to 0.5 indicates a potentially pathogenic variant, whereas higher scores indicate a benign substitution. Conversely, the PMUT and PolyPhen2 platforms use values close to zero indicating a benign variant, with values closer to 1 suggesting a pathogenic variant.

To predict the impact of 5'*-*UTR* LOX* polymorphisms as pathogenic or neutral, the web-based Mutation Taster application (www.mutationtaster.org) was used. PhyloP and phastCons tools were also used to verify the grade of conservation of SNPs. Briefly, phastCons values range from 0 to 1 and reflect the probability that each nucleotide belongs to a conserved regulatory element, based on multiple alignment of genome sequences of 46 different species, as well as alterations in neighboring nucleotides. Values closer to 1 indicate high residue conservation. For phyloP (values ranging from –14 to +6), the same method is used without considering neighboring nucleotides. Positive values are attributed to conserved sites. Additionally, the application predicts the expected impact of altering or creating non-canonical splice sites (including those detected in the *5'-UTR* region of the gene) using an integrated version of the *NNSplice *software*. *Changes in typical splice sites may become stronger or weaker. New splices are indicated by a confidence score higher than 0.3. Information is also provided on the genomic position of the splice site, the prediction scores for reference and mutated sites, and a short sequence indicating the intron/exon border.

In addition, to assess the amino acid conservation, primary amino acid sequences coded for human *SOD1 *and *LOX* genes were aligned to multiple ortholog gene sequences in phylogenetically close organisms downloaded from Ensembl (www.ensembl.org), using the ClustalW Omega software (http://www.ebi.ac.uk/Tools/msa/clustalo/).

##  RESULTS 

Of the 26 patients with advanced KC selected for the present study, 16 (61.5%) were female. Mean age was 28.5 years (SD ± 10.6), and mean KISA% index was 921.5 ( ± 1153.17) for the right eye and 1,451.5 ( ± 607.6) for the left eye. Mean I–S was 6.60 D ( ± 3.13) for the right eye and 6.83D ( ± 3.35) for the left eye. Topography data are summarized in Table 1.

**Table 1:** Mean KISA and I–S indexes in Brazilian patients with keratoconus


**Eye**	**KISA (%)**		**I–S**	
	**Mean**	**SD**	**Mean**	**SD**
Right	921.52	607.6	6.6	3.13
Left	1,451.48	1,153.17	6.83	3.35
KISA, keratoconus percentage index; I-S, inferior-superior value; SD, standard deviation			

**Table 2:** Predicted variant impact on *LOX* protein conformation analyzed by PMUT, SIFT, and PolyPhen2 algorithms. Allelic frequencies are indicated.


**Residue**	**PMUT**	**PolyPhen2**	**SIFT**	**TGP frequency**
R158Q	0.11	0.000	0.21	0.157 (16%)
T392P	0.68	0.087	0.02	ND
TGP, 1000 Genome project; ND, not described			

**Table 3:** Mutation Taster analysis of *SOD1* promoter nucleotide variations


**Variant**	**Effect**	**WT score**	**Variant score**	**Position**	*phyloP*	*phastCons*
–58 C > T	Acc increased	0.43	0.52	g.90	0.216	0.015
–58 C > T	Acc increased	0.24	0.32	g.97	0.216	0.015
–58 C > T	Donor gained	NA	0.38	g.84	0.216	0.015
(–116 C > T)	Acceptor gained	NA	0.32	g.44	1.383	0.058
–58 C > T	Acc increased	0.43	0.52	g.90	0.216	0.015
–58 C > T	Acc increased	0.24	0.32	g.97	0.216	0.015
–58 C > T	Donor gained	NA	0.38	g.84	0.216	0.015
(–116 C > T)	Acceptor gained	NA	0.32	g.44	1.383	0.058

**Table 4:** Supporting Material - PCR Primers and annealing conditions used in this work


**Primer**	**Forward (5'-3')**	** Reverse (5'-3')**	**Tf (bp)**	**Tm (°C)**
LOX – P1a	GAGACTGAGATACCCGTGCT	AGCGGTGACTCCAGATGA	474	58
LOX – P1b	TCACAGTACCAGCCTCAGC	ATAGCTGGGGACCAGGTG	500	60
LOX– P2	TTTTCACATTGCTTTGCAGT	GCTCTTGTCCCACTTCCTAA	398	58
LOX – P3	TAGTTGGGAAAGGAGGATTG	GCAATTTTCTCCCTTCAGGT	355	58
LOX – P4	GACTTATGTCCTGGGGAAAA	GATAAAAATGTGTGTGCTCTTCA	441	58
LOX – P5	GGAGGTGCTATAAGGCTGAG	TTGCTTCCAATACCATGATT	370	58
LOX – P6	TTCAGGGGAAAATATGCAGT	TGCTTACAAGAAAGCTGCTG	394	58
LOX - P7	CTTAGGTGGAGGGAAACTGT	AAGTCATTTTGGCTCATTCA	485	58
SOD1 - P1	CTCCACATTTCGGGGTTCT	ACCCGCTCCTAGCAAAGGT	450	58
SOD1 - P2	CCATCTCCCTTTTGAGGACA	CGACAGAGCAAGACCCTTTC	426	58
SOD1 - P3	TGATGCAGGTCAGCACTTTC	AAAAGCATTCCAGCATTTGG	344	58
SOD1 - P4	CCATCTTTCTTCCCAGAGCA	GAAACCGCGACTAACAATCAA	386	58
SOD1 - P5	TTTGGGTATTGTTGGGAGGA	TCTGTTCCACTGAAGCTGTTT	675	59
SOD1 del	CAGAAACTCTCTCCAACTTTGC	GAGGGGTTTTAACGTTTAGGG	218	59
PCR, polymerase chain reaction			

**Figure 1:**
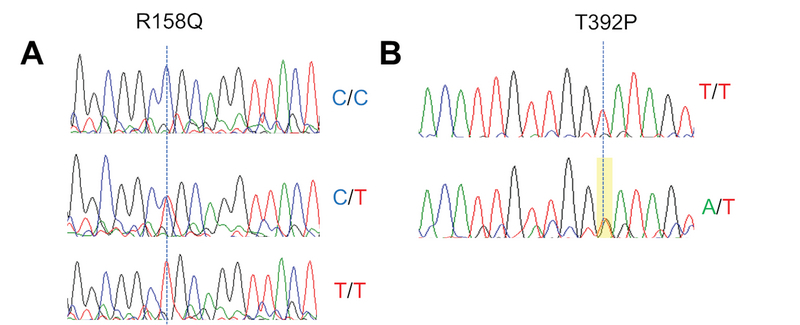
Electropherograms showing R158Q (c.773 C > T) (A) and T392P (c.1474T > A) (B) variants. Genotypes are indicated, and dotted vertical lines indicate peak location for each alteration. Sequencing of T392P variant was obtained using reverse primer.

The sequencing of *LOX* coding region revealed two SNPs. The first variant is a cytosine to thymine transition at position 773 (c.773 C > T) [Figure 1A], observed in three patients – two heterozygotes (C/T) and one homozygote (T/T). This variant leads to replacement of arginine with glutamine at residue 158 (p. Arg158Gln) of the primary *LOX* amino acid sequence. The second variant is an adenine to thymine substitution at position 1474 (c.1474T > A) of *LOX *exon 6 [Figure 1B]. This variant leads to a threonine to proline substitution at residue 392 (p. Thr392Pro) of the primary *LOX* protein sequence. Thr392Pro is a previously unreported mutation of the *LOX *gene. None of these variants were found in control samples.

The assessment of the Thr392Pro mutation using the *PMUT* and *PolyPhen* platforms yielded scores of 0.68 and 0.087, respectively, with 85% probability of pathogenicity [Table 2]. For the Arg158Gln variant, the *PMUT* score was 0.11, indicating a 95% likelihood of this being a neutral mutation, which was confirmed by a low PolyPhen score. The results obtained with SIFT analysis produced scores of 0.02 and 0.21 for Arg158Gln and Thr392Pro, respectively, confirming a deleterious effect of Thr392Pro and a neutral impact of Arg158Gln.

**Figure 2:**
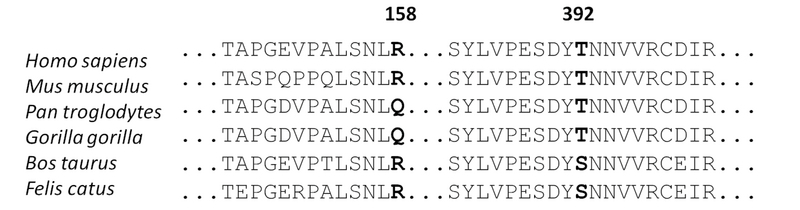
Conservation of *LOX* primary amino acid sequence in several species. Positions of residues R158 and T392 relative to *Homo sapiens* *LOX* protein are indicated.

Regarding the comparison with ancestral residues to determine amino acid conservation across different species through *ClustalW Omega* alignment, the two amino acids were found to be relatively conserved. Threonine at position 392 is greatly conserved in hominids, but not in more phylogenetically distant species [Figure 2].

**Figure 3:**
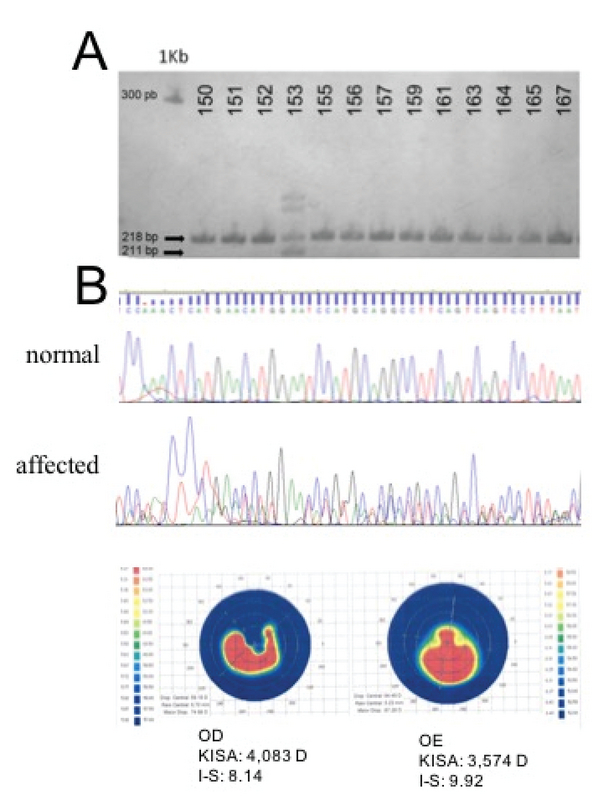
Detection of the IVS2 + 50del7bp deletion in *SOD1* gene in one patient affected with advanced keratoconus. (A) PAGE electrophoresis showing a subset of patients tested for the deletion (numbers indicated above). Amplicons of 218 pb (normal) and 211 pb (deleted) fragments are indicated by black arrows. A 1 kb DNA ladder shows the 300 pb reference fragment (smaller fragments are not shown). (B) Electropherograms of the deletion region from normal and affected patients. (C) Corneal topographies of KC patient carrying the *SOD1* deletion. PB, base pairs; KB, kilobases; OD, right eye; OE, left eye.

*SOD1* intronic 7-base deletion was detected in one patient [Figure 3A]. The patient was diagnosed with advanced KC, presenting KISA values of 4,083 and 3,574 diopters and I–S values of 8,14 and 9,92, for right and left eyes, respectively. This variant was not detected in any of the non-affected individuals. The sequencing of this region in the carrier showed an electropherogram pattern that suggests a concatenated overlapping of the wild-type and deleted alleles, a typical pattern observed in individuals who are heterozygote for deletion [Figure 3B].

**Figure 4:**
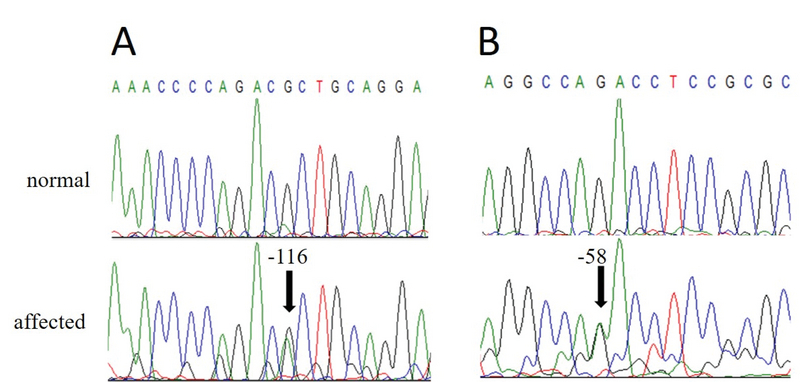
Electropherograms showing 5'UTR polymorphisms located at positions –116C > T (A) and –58C > T (B) relative to *SOD1* ATG start site. Sequencing was performed using reverse primer.

**Figure 5:**
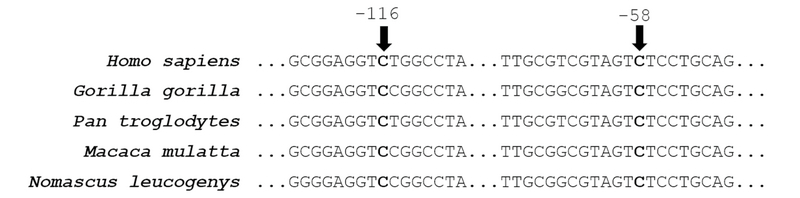
DNA sequence alignment of *SOD1* 5'UTR sequences from different species. Conserved positions of residues –116 and –58 relative to *Homo sapiens* sequence are indicated.

In addition, we observed the presence of two base polymorphisms at the untranslated 5' region (5'-UTR) of *SOD1* in two affected individuals. The two variants involved replacement of thymine with cytosine, one at position –116 (–116C > T) and the second at position –58 (–58C > T), both located at the *SOD1* promoter region. Electropherograms for these variants showed heterozygous point mutations in both patients [Figure 4]. Variant –58C > T had already been described in the dbSNP (rs906695375) databank, but not in 1000 Genomes. In the present work, this variant was detected in a proband with KISA score of 1,294% and I–S of 4.68 in the right eye and KISA of 2,592% and I–S score of 7.24 in the left eye. No other variants were found in *SOD1* coding region in the 26 patients analyzed. The alignment of the human *SOD1* promoter region sequence with that of other hominids show conservation of these residues over evolution [Figure 5].

An analysis of the impact of variants in the *SOD1 *gene promoter region showed a significant conservation score (0.216) with phyloP analysis for the –58C > T variant, as well as for the upstream (0.5) and downstream (1.771) flanking region phastCons analysis, which also showed significance for this variant, with values slightly higher than zero [Table 3]. A positive modulation by variant –58C > T of the splice acceptor sites predicted at positions 90 and 97 of the gene, with scores of 0.52 and 0.32, respectively, was suggested. In addition, the analysis suggests the creation of a new ectopic splice donor site at position 84, with a score of 0.38. Analysis of variant –116C > T showed a new splice acceptor site at position 44, with a score of 0.32. Significant conservation scores were also observed for phyloP (1.383) and phastCons (0.058).

##  DISCUSSION

Cornea has been the most transplanted tissue in Brazil since the late 1990s, with KC among the main etiologies.^[39,40]^ Molecular and linkage studies have suggested multiple candidate genes that might underlie the development of KC.^[32]^ In the present work, we found two non-synonymous mutations in *LOX*, namely Arg158Gln and Thr392Pro, and an intronic *SOD1* deletion previously associated with KC development. Variant Thr392Pro in the *LOX* gene is a novel missense mutation that has not been previously described; this mutation was found in 1 out of 26 patients with advanced KC. The scores obtained with computational algorithms suggest a deleterious impact of this substitution on protein stability resulting from the replacement of threonine (aliphatic open side chain) with proline. Proline is an amino acid with non-polar side chain, therefore not capable of ionization and hydrogen bonding, and featuring a secondary rather than a primary amino group. This suggests that the substitution may possibly affect protein folding and compromise cellular function. In addition, threonine was conserved in hominids, which supports the notion that this residue is important for normal *LOX* function. Nevertheless, the non-conserved amino acid in *Felis catus* and *Bos taurus *is a serine, which presents a side chain with similar biochemical properties (both containing aliphatic hydroxyl groups), indicating an essential role in the secondary *LOX* structure of all species considered.

We found the missense Arg158Gln variant in *LOX* gene, detected in 3 of the 26 patients analyzed, with one being homozygous and two heterozygous for this mutation. This substitution has also been described (rs1800449) in other ethnic groups.^[16,20]^ However, a meta-analysis did not find sufficient evidence in the literature to associate this SNP with the development of KC.^[15]^ In fact, *in silico *assessments performed with PMUT, SIFT, and PolyPhen-2 algorithms produced scores indicating this as a neutral, non-pathogenic substitution. This finding is confirmed by the high frequency of the T allele, found in 16% of the world population according to the 1000 Genome Project database. In addition, the glutamine for arginine (both of which have open chains of similar size) substitution is well-tolerated because of the similar physicochemical characteristics of these amino acids and does not substantially interfere with *LOX* protein folding. The alignment of *LOX* sequences of phylogenetically close species also revealed non-conservation of this arginine. Therefore, the present findings suggest low impact of the Arg158Gln variant on LOX function.

For the first time, we identified the IVS2 + 50del7bp deletion in *SOD1 *gene in the Brazilian population. This variant was detected in 1 patient with severe KC out of 140 KC affected volunteers, which was absent in 120 healthy controls. The literature has described significant association of this deletion with KC development both in familial and sporadic cases of various ethnicities. A study conducted by Udar et al reported a haplotype-independent segregation of this deletion in two non-related American families presenting an autosomal dominant form of KC.^[27]^ This finding suggests a likely autonomous effect of *SOD1* IVS2 + 50del7bp on KC pathogenesis. An mRNA analysis of *SOD1* carrying this deletion showed, in addition of the wild-type transcript, two splice variants lacking exon 2, or both exon 2 and 3 simultaneously, which exclude *SOD1* protein active site and lead to a loss of function protein.^[27]^ Another study with 33 Greek patients found that homozygote carriers for this deletion were significantly overrepresented among KC patients compared to healthy subjects.^[29]^ We also verified a similar frequency of IVS2 + 50del7bp allele found with other studies.^[30]^ We believe that this specific *SOD1* deletion, yet with a low frequency in all cohorts studied, reinforces IVS2 + 50del7bp deletion as a key genetic alteration highly associated with KC in different populations.

We also detected two SNPs in the untranslated 5' region of *SOD1*, both characterized by replacements of cytosine with thymine at positions –58 and –116 in two different patients. There have been no reports in the literature of any of the two variants described in the present work. *In silico* analysis showed scores indicating increased activity of splice acceptors in the presence of variant –58C > T. In addition, these scores also showed high probability of these variations having a pathological impact on *SOD1* activity because of the creation of donor (–58C > T) and acceptor (–116C > T) splice sites. This is a noteworthy finding, since alterations in the *SOD1* promoter region sequence may cause a decrease in protein expression, as shown for other disorders.^[41,42]^ The *SOD1* protein neutralizes excess ROS in the cell, and ROS levels are increased in patients with KC.^[43,44,45]^ Low levels of *SOD1* enzyme can also result from gene mutations.^[28]^ It is possible that these variants may be related to changes in the function of this gene in patients with KC, considering that these are highly conserved residues and the predicted splice sites may influence the normal processing points at the exon–intron boundaries in the gene. We believe that these 5'-UTR variants may modulate *SOD1* gene expression in KC patients, although additional *in vitro* studies involving cloning of the *SOD1* gene, with a focus on the variants described in the present study and analysis of the impact of these variants on transcription rates, are essential to confirm the involvement of these polymorphisms in the pathophysiology of KC.

In conclusion, we report a novel Thr392Pro with potential damaging effect to *LOX* protein in a Brazilian patient presenting advanced KC, as well as a previously described Arg158Gln variant. We also detected a previously KC associated *SOD1 *IVS2 + 50del7bp deletion in another patient with severe KC, and two SNPs located in* SOD1* 5'UTR with still unclear involvement in KC pathogenesis. *SOD1* and *LOX* variants found in Brazilian unrelated KC patients suggests a genetic contribution to disease onset and progression, although further studies will be key to validate their impact on gene transcription and protein function. In addition, variant screening in larger samples will be essential to evaluate the real contribution of these polymorphisms to KC pathophysiology in Brazilian population.

##  Acknowledgements

The authors are grateful to Iracema Freitas da Silva for technical support and Fabio Mendes da Silva for blood sampling and topography imaging. The equipment and reagents acquisition were funded by CESED/UNIFACISA institutional resources.

##  Financial Support and Sponsorship

Nil.

##  Conflicts of Interest

There are no conflicts of interest.
